# Dental, skeletal and soft tissue effects of the Distal Jet appliance: A prospective clinical study

**DOI:** 10.1590/2177-6709.24.6.056-064.oar

**Published:** 2019

**Authors:** Rachelle Simões Reis, José F. C. Henriques, Guilherme Janson, Karina Maria Salvatore Freitas, Wilana Moura

**Affiliations:** 1 Universidade de São Paulo, Faculdade de Odontologia de Bauru, Departamento de Ortodontia (Bauru/SP, Brazil).; 2 Centro Universitário Ingá, Departamento de Ortodontia (Maringá/PR, Brazil).

**Keywords:** Orthodontic appliance, Orthodontics, corrective, Malocclusion, Angle Class II

## Abstract

**Objective:**

This study evaluated the dental, skeletal and soft tissue effects in Class II malocclusion patients treated with Distal Jet appliance, compared to an untreated control group.

**Methods:**

44 patients with Class II malocclusion were divided into two groups: Group 1 (experimental) - 22 patients, mean age of 12.7 years, treated with the Distal Jet appliance for a mean period of 1.2 years; Group 2 (control) - 22 untreated patients, mean age of 12.2 years, followed by a mean period of 1.2 years. Lateral cephalograms were obtained before treatment (T_0_) and at the end of the distalization (T_1_).Independent *t* test was used to identify intergroup differences.

**Results:**

When compared to control group, the Distal Jet produced a significant increase in mandibular plane angle (0.7 ± 2.0^o^). The maxillary second molars presented distal inclination (6.6 ± 3.8^o^), distalization (1.1 ± 1.1 mm) and extrusion (1.3 ± 2.1 mm). The maxillary first molars distalized by 1.2 ± 1.4 mm. The maxillary first premolars mesialized by 3.4 ± 1.1 mm. The maxillary incisors showed slight labial tipping of 4.3 ± 4.7^o^ and were protruded by 2.4 ± 1.7 mm. There were no significant changes in the facial profile. The overjet increased 1.5 ± 1.1 mm and overbite had no significant changes.

**Conclusion:**

The Distal Jet appliance is effective to distalize the maxillary first molars, but promotes increase in mandibular plane angle, distal inclination, extrusion and distalization of maxillary second molars, mesialization of maxillary first premolars, proclination and protrusion of maxillary incisors, and increase in overjet, when compared to a control group.

## RESUMO

**Objetivo:**

o presente estudo avaliou os efeitos dentoesqueléticos e tegumentares em pacientes com má oclusão de Classe II tratados com aparelho Distal Jet, comparando-os com um grupo controle não tratado.

**Métodos:**

44 pacientes com má oclusão de Classe II foram divididos em dois grupos: Grupo 1 (experimental) - 22 pacientes, idade média de 12,7 anos, tratados com o aparelho Distal Jet por um período médio de 1,2 anos; Grupo 2 (controle) - 22 pacientes não tratados, idade média de 12,2 anos, acompanhados por um período médio de 1,2 anos. Telerradiografias laterais foram obtidas antes do tratamento (T_0_) e no final da distalização (T_1_). O teste *t* independente foi usado para identificar as diferenças entre os grupos.

**Resultados:**

quando comparado ao grupo controle, o Distal Jet produziu um aumento significativo no ângulo do plano mandibular (0,7 ± 2,0^o^). Os segundos molares superiores apresentaram inclinação distal (6,6 ± 3,8^o^), distalização (1,1 ± 1,1 mm) e extrusão (1,3 ± 2,1 mm). Os primeiros molares superiores foram distalizados por 1,2 ± 1,4 mm. Os primeiros pré-molares superiores, mesializados por 3,4 ± 1,1 mm. Os incisivos superiores mostraram leve inclinação labial de 4,3 ± 4,7^o^ e foram protruídos por 2,4 ± 1,7 mm. Não existiram alterações significativas no perfil facial. O *overjet* aumentou 1,5 ± 1,1 mm, e o *overbite* não sofreu alterações significativas.

**Conclusão:**

o aparelho Distal Jet é eficaz para distalizar os primeiros molares superiores, mas promove aumento no ângulo do plano mandibular, inclinação distal, extrusão e distalização dos segundos molares superiores, mesialização dos primeiros pré-molares superiores, vestibularização e protrusão dos incisivos superiores e aumento do *overjet*, quando comparado a um grupo de controle.

## INTRODUCTION

There are several mechanisms for molar distalization, including intraoral distalizer appliances such as the Distal Jet appliance. These appliances can be used with conventional and skeletal anchorage.^1^ ^-^ ^6^ According to Cozzani et al,^7^ when the Distal Jet with skeletal anchorage was compared with the Distal Jet with conventional anchorage, the mean molar distalization and treatment time were similar.

Skeletal anchorage devices have easy installation and can be used with intraoral distalizer to control undesirable side effects. However, these devices cannot be used in all cases, for several factors as failure of the miniscrews, no patient acceptance due to surgical procedure necessary to installation, or by the presence of systemic disorder.^8^ ^-^ ^11^ In these cases, the orthodontist should use intraoral distalizer with conventional anchorage.

Kinzinger et al^12^ performed a systematic review to compare the efficiency of various appliance types with intramaxillary anchorage for molar distalization. The authors observed that the First Class and the Distal Jet appliances are more efficient than the Jones Jig appliance for molar distalization. 

The mechanisms for molar distalization with intraoral conventional anchorage are considered a practical resource for anteroposterior correction, since these appliances promote more space gain in the maxillary arch and correct the Class II molar relationship with reduced need of patient compliance.^6^ ^,^ ^13^ ^-^ ^18^

The dental, skeletal and soft tissue effects of Distal Jet appliance were evaluated by some studies,^16^ ^-^ ^21^ but only the study of Vilanova et al^21^ compared the effects with a control group; however, the study evaluated the effects of the Distal Jet appliance followed by fixed appliances, thus more studies are necessary. Therefore, this study aimed to evaluate the dental, skeletal and soft tissue effects in treatment of Class II malocclusion with the use of the Distal Jet appliance, comparing to an untreated control group. 

## MATERIAL AND METHODS

### Material

This prospective study was approved by the ethics in research committee of Bauru Dental School, University of São Paulo, Brazil (protocol number: 1462004). The parents or legal guardians of all patients signed an informed consent allowing treatment to be performed.

The sample size was calculated based on an alpha significance level of 0.05 and a beta of 0.2 to achieve 80% of power to detect a mean difference of 1.6mm with a standard deviation of 1.84 in the 6-PTV changes after the distalization with the Distal Jet appliance, between the groups.^22^ The sample size calculation showed that 22 patients were needed in each group.

The sample consisted of 44 patients with Class II malocclusion divided into two groups. Group 1 (experimental) - 22 patients (5 male; 17 female), at a mean age of 12.7 years (SD = 1.2, range 10.5 to 14.7 years), treated with the Distal Jet appliance. These patients were prospectively treated at the Bauru Dental School, by the same orthodontist. All patients presented the germen of the maxillary third molars and the maxillary second molars erupted at the beginning of treatment. Four patients presented ¼ cusp Class II molar relationship, 16 presented ½ cusp Class II and 2 presented – cusp Class II molar relationship. Patients of Group 1 were selected in Orthodontic Department of Bauru Dental School to treatment according to the following inclusion criteria.

» Class II malocclusion, division 1 or 2.
» Absence of transversal discrepancies.
» Minimal or no crowding in the mandibular arch.
» Permanent dentition.
» FMA angle smaller than 31^o^.
» Non-extraction treatment.
» Balanced facial profile.

Group 2 (control) - 22 subjects (11 male; 11 female), at a mean age of 12.2 years (SD = 0.8, range 11.0 to 14.7 years) obtained from the files of the same Orthodontic Department, who did not receive any orthodontic treatment. The control group was an untreated historical control group selected in order to match the experimental group, according to the inclusion criteria. Initial ages and observation time were matched between the groups. Besides, sex distribution in the groups was also matched. The type of malocclusion was Class II, the same as the experimental group, and the severity of this malocclusion was also matched between the two groups. Good-quality radiographs with adequate landmark visualization were used to select the Group 2. In control group, all patients presented the germen of the maxillary third molars, too.

**Figure 1:**
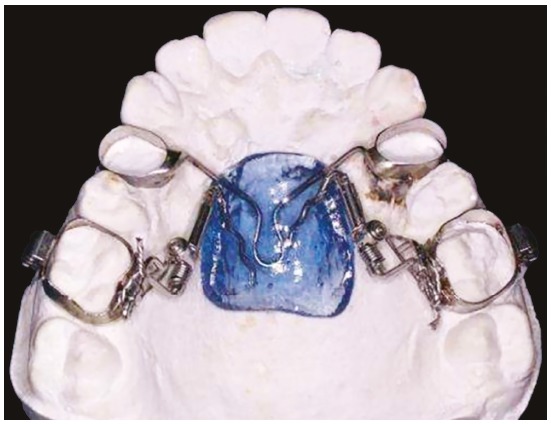
Distal Jet appliance.

All patients in the experimental group received a Distal Jet appliance (American Orthodontics, Sheboygan, Wisconsin) proposed by Carano and Testa^23^ (Fig 1).Bands were fitted on the maxillary first molars and maxillary first premolars, and then the maxillary dental casts were sent to the laboratory for appliance construction. Coil springs were activated every 4 to 6 weeks. The forces generated by the NiTi coils were of 240g.

**Figure 2:**
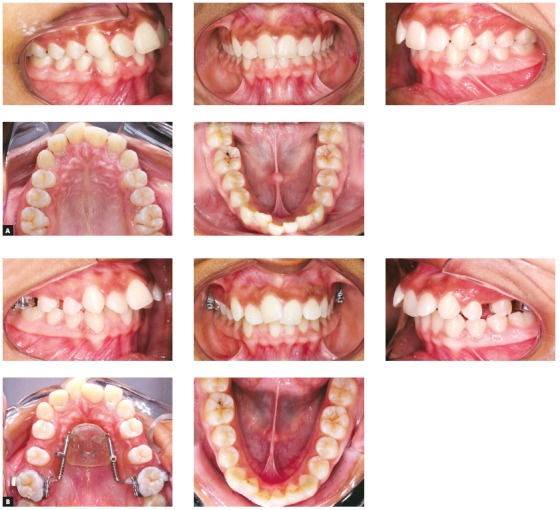
Maxillary molar distalization and final results after use Distal Jet appliance: A) initial; B) final.

Eighty-eight lateral cephalograms were taken for the study. Cephalometric headfilms were collected at the beginning (T_0_) and at the end of the Distal Jet appliance treatment (T_1_). The mean time period between the initial T_0_ radiograph and the post-treatment T_1_ radiograph was 1.2 years (SD = 0.3, range 0.6 to 1.6 years). Figure 2 illustrates a maxillary molar distalization and final results after use of the Distal Jet appliance.

### Statistical analyses

Lateral cephalograms were evaluated after one month interval and casual and systematic error study was performed. Comparability between the groups regarding sex distribution and malocclusion severity were performed with chi-square tests and comparability of initial and final ages, treatment/observation periods and pretreatment cephalometric variables were performed with independent *t* tests. Intergroup changes of each variable from T_0_ to T_1_ were statistically analyzed with independent *t* tests. 

Statistical analyses were performed with Statistica for Windows software (version 6.0, StatSoftInc, Oklahoma, USA). Differences with a probability of error less than 5% (*p*< 0.05) were considered statistically significant.

## RESULTS

The casual errors varied from 0.28 mm (E-LL) to 0.77 mm (Co-Gn) and from 0.33^o^ (SN.Gn) to 2.61^o^ (NLA), and only four of the thirty variables presented systematic errors (SNB, 7-PTV, 7-PP, 4-PP). 

The groups were comparable regarding initial and final ages, treatment/observation period, sex distribution and malocclusion severity. Cephalometrically, at pretreatment only two variables (7-PTV - higher in experimental group and 6.SN - higher in control group) were significantly different between the groups (Tables 1 and 2). 

**Table 1:** Comparability between the groups.

Variables (years)	Group 1 (Experimental)		Group 2 (Control)		p value	
	Mean	SD	Mean	SD		
Initial age	12.7	1.2	12.2	0.8	0.145^t^	
Final age	13.9	1.2	13.4	0.7	0.139^t^	
Observation time	1.2	0.3	1.2	0.3	0.986^t^	
Sex	Group 1 (Experimental)		Group 2 (Control)		P	X^2^
Male	5		13		0.06^c^	3.54
Female	17		9			
Malocclusion severity	Group 1 (Experimental)		Group 2 (Control)		P	X^2^
¼ Cl II	4		5		0.80^c^	0.44
½ Cl II	16		14			
– Cl II	2		3			

“Statistics: t, test t independent; c, chi-square test.”

**Table 2:** Comparison initial cephalometric values between groups.

Variables	Group 1		Group 2		p Value
	Mean	SD	Mean	SD	
SNA	81.7	4.1	82.0	2.9	0.788
A-PTV	47.8	2.4	48.9	1.9	0.098
SNB	77.6	2.9	78.3	3.2	0.411
B-PTV	46.2	4.5	46.9	4.1	0.596
Go-Gn	70.3	3.7	71.6	3.7	0.263
Co-Gn	104.2	4.4	106.3	4.7	0.143
ANB	4.1	2.2	3.6	2.0	0.478
NAP	6.2	5.6	5.6	5.3	0.754
FMA	26.2	3.5	26.2	3.7	0.996
SN.GoGn	31.6	3.6	31.1	4.9	0.668
LAFH	60.4	4.5	60.7	3.3	0.222
SN.PP	6.6	2.4	7.3	3.9	0.094
NS.GN	67.0	2.9	66.5	3.4	0.979
7.SN	63.5	4.2	63.2	4.9	0.851
7-PTV	12.1	2.5	10.6	1.9	0.029*
7-PP	11.4	3.4	10.3	2.8	0.232
6.SN	72.6	4.0	75.8	4.4	0.016*
6-PTV	21.2	3.0	20.1	2.4	0.190
6-PP	16.4	2.1	16.4	11.4	0.980
4.SN	83.8	5.3	82.3	5.0	0.335
4-PTV	36.3	3.2	35.3	2.2	0.271
4-PP	19.7	2.3	19.1	2.0	0.359
1.SN	104.2	7.3	104.1	5.9	0.964
1-PTV	54.7	4.2	55.0	2.6	0.815
1-PP	26.7	2.7	26.3	2.3	0.674
NLA	105.2	13.9	109.5	10.2	0.251
UL-E	2.4	2.5	2.1	2.2	0.668
LL-E	0.4	2.3	1.0	1.9	0.361
Overjet	4.8	1.5	5.1	1.8	0.684
Overbite	4.0	1.9	4.4	1.4	0.483

*Statistically significant at p < 0.05.

Skeletal assessments showed that the cranial base remained constant in both groups. The mandibular plane angle increased significantly in experimental group (0.7 ± 2.0^o^) and reduced in control group (-0.7 ± 1.5^o^). In experimental group, the maxillary second molars showed significant distal inclination (-6.6 ± 3.8^o^), distalization (-1.1 ± 1.1 mm) and extrusion (1.3 ± 2.1 mm), while in control group, the maxillary second molars showed mesial inclination (1.6 ± 5.2^o^), mesialization (0.9 ± 1.8 mm) and extrusion (2.9 ± 1.5 mm) (Table 3).

**Table 3:** Means and standard deviations of cephalometric changes after distalization (T0-T1).

Variables	Group 1 (Experimental)		Group 2 (Control)		P value
	Mean	SD	Mean	SD	
SNA	0.0	2.0	0.0	1.4	0.899
A-PTV	0.4	1.7	1.0	1.3	0.203
SNB	0.4	1.4	-0.1	0.9	0.121
B-PTV	1.0	2.0	0.9	2.1	0.853
Go-Gn	1.3	1.5	1.6	1.3	0.513
Co-Gn	2.1	2.9	2.1	2.3	0.968
ANB	-0.3	1.4	0.1	0.8	0.150
NAP	-1.0	3.1	0.0	2.0	0.173
FMA	0.7	2.0	-0.7	1.5	0.006*
SN.GoGn	-0.0	1.4	0.1	1.2	0.647
LAFH	2.2	1.8	1.5	1.8	0.222
SN.PP	0.0	1.5	0.7	1.4	0.094
NS.GN	0.2	1.2	0.2	1.0	0.979
7.SN	-6.6	3.8	1.6	5.2	0.000*
7-PTV	-1.1	1.1	0.9	1.8	0.000*
7-PP	1.3	2.1	2.9	1.5	0.007*
6.SN	-2.4	5.2	0.1	4.4	0.079
6-PTV	-1.2	1.4	1.1	1.6	0.000*
6-PP	0.6	1.8	1.0	1.2	0.495
4.SN	-0.3	3.3	-0.8	2.8	0.597
4-PTV	3.4	1.1	0.9	1.6	0.000*
4-PP	1.6	1.4	0.8	1.1	0.070
1.SN	4.3	4.7	-0.3	3.0	0.000*
1-PTV	2.4	1.7	1.0	1.4	0.004*
1-PP	0.4	1.2	0.4	0.8	0.954
NLA	1.7	15.5	0.9	7.2	0.842
UL-E	0.1	1.3	0.5	0.7	0.235
LL-E	-0.0	1.4	0.5	0.8	0.105
Overjet	1.5	1.1	-0.0	0.7	0.000*
Overbite	-0.4	1.3	-0.2	1.2	0.542

*Statistically significant at p < 0.05.

In this study, the experimental group showed significant distalization of the first molars by a mean of 1.2 ± 1.4 mm and the control group showed mesialization by a mean of 1.1 ± 1.6 mm. The maxillary first premolars mesialized in both groups, but in experimental group the mesialization was greater (3.4 ± 1.1 mm). The maxillary incisors showed labial tipping of 4.3 ± 4.7^o^ in experimental group and slight lingual tipping of 0.3 ± 3.0^o^ in control group. In both groups, the maxillary incisors were protruded, but the protrusion was grater in experimental group (2.4 ± 1.7 mm). There were no significant changes in the facial profile. The overjet increased significantly 1.5 ± 1.1 mm in experimental group and practically did not change in the control group (0.0 ± 0.7 mm). Overbite in both groups showed no significant changes (Table 3).

## DISCUSSION

The strength of this study is the use of a control group, showing not only the effects with the Distal Jet appliance, but comparing it to a matched control group. The groups were comparable regarding initial and final ages, treatment/observation periods, sex distribution, malocclusion severity and initial cephalometric characteristics (Tables 1 and 2). The present sample included mostly patients with half-cusp Class II malocclusion, while most studies on the Distal Jet appliance did not mention the Class II malocclusion severity of their patients.^17^ ^,^ ^18^ Therefore, this has to be considered in the comparison to other studies.

The number of the patients could be greater. The initial experimental sample treated prospectively included 30 patients. However, some of them gave up the orthodontic treatment or moved out to other cities. And when the experimental and control samples were selected and matched, it was necessary to remove some patients from the sample, in order to make them comparable.^24^ ^,^ ^25^

There were no significant changes on the sagittal position of the maxillary and mandibular apical bases and in the maxillary-mandibular relationship, demonstrating that the Distal Jet does not influence the anteroposterior behavior of the skeletal bases. The skeletal results were already expected because the Distal Jet does not have skeletal effects, as found by others studies.^6^ ^,^ ^16^ ^,^ ^17^ ^,^ ^26^ Only the mandibular plane angle (FMA) showed a statistically significant increase in the experimental group, when compared to the control, but this change is temporary (Table 3). A possible explanation to the increase of FMA is the side-effect caused by extrusion, distalization mechanics and tipping of the maxillary second molars. When compared with control group, the maxillary second molars were less extruded, but these teeth were also distalized, therefore the FMA angle increased.

According to Kinzinger et al,^27^ the presence of the germ of the maxillary third molars in patients may influence the increase in the tipping of the second molars and consequently the increase of the FMA. However, the study of Vilanova et al^21^ did not find significant increase of the FMA after treatment with Distal Jet followed by fixed appliance, but found significant increase of the SN.occlusal plane angle, though this alteration can be due to the use fixed appliances. 

However, in this study, it was observed that a greater extrusion of the second molar was found in the control group and that this extrusion did not cause increase in the FMA on this group, contrary to the expected. The extrusion in control group was accompanied by mesial movement due to normal growth development, and this could explain why the FMA did not increase in the control group. Also, the change in the mandibular plane angle can be related with other factors such as facial growth pattern and the mandibular growth pattern of the patient. Therefore, more studies are necessary to identify the vertical skeletal alterations resulting of the treatment with Distal Jet. 

The first molars showed a slight non-significant distal tipping, but a greater amount of distal tipping was observed in the second molars, when compared to the control group. The greater amount of distal tipping observed in the second molars could be due to the developmental stage of the third molars, which could influence the amount of tipping of the second molars. This is because, if the germ of the third molar is above the center of resistance of the second molar, it works as a rotation fulcrum and tends to distally tip the second molar.^14^ Besides, the entire sample presented the maxillary second molars erupted at the beginning of treatment, avoiding the excessive distal tipping of maxillary first molars.^27^ ^,^ ^28^

The maxillary second molars were moved distally 1.1 mm in the experimental group, while the control group showed a mesial movement of 0.9 mm, which represents a 2.0-mm correction in the Class II relationship (Table 3). There was 1.2 mm of maxillary first molar distalization in the experimental group, while the control group showed a mesial movement of 1.1 mm, which represents a 2.3-mm correction in Class II molar relationship (Table 3). A mesial movement in control group results from the growth of patients during the follow-up period.

These results were similar in other studies^16^ ^,^ ^17^ ^,^ ^29^ however, with a relatively smaller distalization, probably because all patients presented the germs of third molars, making the distalization difficult. The amount of molars distalization of this study was small. However, Ngantung et al^16^ and Nishii et al^26^ found similar results. Bolla et al^17^ found greater molar distalization than the present study. However, none of these studies mentioned the presence or not of erupted maxillary second molars, which prevents a direct comparison with the present results. 

The results of this study also showed a significant but small distalization of the maxillary molars. This reduced amount of distalization was caused by the presence of the germs of third molars, and the presence of erupted second molars, making the distalization more difficult. When treating patients with the Distal Jet appliance, one should observe the presence or not of the second and third molars.^27^ ^,^ ^30^ If more distalization is needed, it is indicated to use the appliance early, when the second molars are not erupted. If the tipping should be avoided, the presence of the second and third molars is desired. These characteristics should be observed in order to obtain the best results for each individual case to be treated. 

According to Bolla et al,^17^ there is influence in the total space created during distalization of the anterior teeth anchorage loss. Thus, a relatively smaller distalization can be associated with the anchorage loss in the anterior teeth because the force used to distalize is dissipated in a mesial movement of the anterior teeth. 

There was significant mesial movement of maxillary premolars, when compared to the control group (Table 3). There was significant protrusion and labial tipping of the maxillary incisors that were protruded by 2.4 ± 1.7 mm and showed slight labial tipping of 4.3 ± 4.7^o^. There was no significant change in the soft tissue component in the group treated with the Distal Jet. There was an increase of 1.5 ± 1.1 mm in overjet in the experimental group, when compared to the control (Table 3). These results were also observed in other studies that used intraoral distalizers, including Distal Jet.^6^ ^,^ ^13^ ^-^ ^18^ ^,^ ^23^ ^,^ ^26^ ^,^ ^31^ ^,^ ^32^

There were no significant changes in the overbite and facial profile (Table 3). However, some studies showed that anchorage loss can influence the position of maxillary incisors and may cause changes in the profile.^15^ ^,^ ^31^ ^,^ ^32^

Intraoral distalizer effects with conventional and skeletal anchorage were studied in systematic review performed in 2013.^6^ This review concluded that both anchorage systems (conventional and skeletal) are effective for distalization, with skeletal anchorage presenting lower loss of anchorage. Therefore, the use Distal Jet appliance for the treatment of the Class II malocclusion is a viable alternative, especially when some effects of the appliance can favor the treatment. For example, in Class II, division 2 malocclusion, the maxillary incisors need to be protruded and distal jet appliance promotes this protrusion, or when the miniscrew cannot be used.

Other two systematic reviews were performed to evaluate the quantitative effects of the conventional pendulum appliance and modified pendulum appliance and of miniscrew-supported appliances for maxillary molar distalization in Class II malocclusions.^33^ ^,^ ^34^ However, both studies present simple descriptive and stratified comparisons and reported limitations, due to heterogeneity among the studies, that impaired the realization of meta-analysis. 

Currently, there are several orthodontic distalization appliances. Studies like this are important for the orthodontist to know and understand the effects of a specific appliance. 

## CONCLUSIONS

The use of Distal Jet appliance compared to a control group can cause increase in mandibular plane angle, distal inclination, distalization and extrusion of the maxillary second molars, distalization of the first molars, mesialization of the maxillary first premolars, and labial inclination and protrusion of the maxillary incisors, leading to an increase in the overjet.
